# Equol, a Clinically Important Metabolite, Inhibits the Development and Pathogenicity of *Magnaporthe oryzae*, the Causal Agent of Rice Blast Disease

**DOI:** 10.3390/molecules22101799

**Published:** 2017-10-24

**Authors:** Jiaoyu Wang, Ling Li, Yeshi Yin, Zhuokan Gu, Rongyao Chai, Yanli Wang, Guochang Sun

**Affiliations:** 1 State Key Laboratory Breeding Base for Zhejiang Sustainable Pest and Disease Control, Institute of Plant Protection and Microbiology, Zhejiang Academy of Agricultural Sciences, Hangzhou 310021, China; liling-06@163.com (L.L.); yinyeshi@126.com (Y.Y.); gzkcpz@163.com (Z.G.); rychai@sina.com (R.C.); ylwang88@aliyun.com (Y.W.); 2 The Key Laboratory for Quality Improvement of Agricultural Products of Zhejiang Province, School of Agricultural and Food Sciences, Zhejiang Agriculture and Forest University, Hangzhou 311300, China

**Keywords:** equol, antifungal activity, plant pathogen, fungal pathogenicity, *Magnaporthe oryzae*

## Abstract

Equol, a metabolite of soybean isoflavone daidzein, has been proven to have various bioactivities related to human health, but little is known on its antifungal activity to plant fungal pathogens. *Magnaporthe oryzae* is a phytopathogenic fungus that causes rice blast, a devastating disease on rice. Here, we demonstrated that equol influences the development and pathogenicity of *M. oryzae*. Equol showed a significant inhibition to the mycelial growth, conidial generation and germination, and appressorial formation of *M. oryzae*. As a result, equol greatly reduced the virulence of *M. oryzae* on rice and barley leaves. The antifungal activity of equol was also found in several other plant fungal pathogens. These findings expand our knowledge on the bioactivities of equol.

## 1. Introduction

Equol (4,7-isoflavandiol) was discovered by Marrian et al. in 1932 when they separated hydroxyestrone from pregnant mare urine, and determined its molecular formula as C15H1403. It was assigned the name equol because it is a metabolic product of equine and belongs to the diphenol with estrogen action [1]. Equol exists in two enantiomeric forms, (*S*)-equol and (*R*)-equol [2]. (*S*)-equol can be converted from soy isoflavone daidzein in humans and animals after soy isoflavone consumption based on the presence of certain bacteria in their gastrointestinal tracts [2,3]. Approximately 30–50% of humans are capable of producing (*S*)-equol [4]. In contrast, (*R*)-equol is not made in humans, but can be chemically synthesized in the laboratory [5].

In recent years, equol has received considerable attention due to its diverse biological activities, including antioxidant and antitumor properties [6,7,8]. Based on these studies, (*S*)-equol is a potential nutraceutical agent possessing significant health benefits. Despite its safety and adverse effects still being held under suspicion by the medical and scientific community, (*S*)-equol and its related compounds have been used in many dietary supplements for their potential protective effects against aging, skin conditions, hair loss, prostate cancer, obesity, hot flashes, menopause, osteoporosis, heart disease, and neurologic conditions [9,10]. However, these studies rarely concerned the antimicrobial activity of equol. It is still obscure whether and how equol affects the growth and development of fungal pathogens.

*Magnaporthe oryzae*, a heterothallic ascomycete fungus, causes rice blast, the most devastating disease on rice, and also infects many other economically important cereal crops, such as barley, oats, rye grass, and millets [11]. *M. oryzae* uses its conidia to infect hosts and disseminate. Once the conidium of *M. oryzae* lands on the plant surface, it germinates within 2 h. The germ tube is induced by specific physical and chemical factors, such as a hydrophobic plant surface, to swell on its top and differentiate into a type of specialized infective structure, called an appressorium. The appressorium accumulates a high concentration of glycerol and generates enormous internal turgor [12], and on the leaf surface, this turgor converts into a high mechanical force. Subsequently, the fungus elaborates a slender hypha, namely the penetration peg, on the bottom of the appressorium. With the high mechanical force, the penetration peg breaches the plant cuticle and invades the underlying epidermal cells [13]. Finding molecules that possess an inhibition effect to the development of *M. oryzae*, especially to appressorial formation and pathogenicity, would greatly benefit the control of the rice blast disease.

To explore new bioactivities of equol and also find potential molecules to control plant diseases, in the present work, we analyzed the antifungal activity of equol against *M. oryzae* at different development stages.

## 2. Materials and Methods

### 2.1. Fungal Strains and Growth Conditions

The *M. oryzae* Guy-11 strain was used [14]. The *MoPEX5* and *MoPEX7* deletion mutants were both derived from Guy-11 [15]. The *Botrytis cinerea* used was strain B05.10 [16]. The *Alternaria alternata* used was Nt18, a strain isolated from tobacco (*Nicotiana tabacum*) with brown spot disease in Yunnan province and identified by its biological characters and internal transcribed spacer (ITS) sequences. The *Colletotrichum fragariae* used was the ZJ91 strain isolated from strawberry (*Fragaria* × *ananassa*) with anthracnose in Zhejiang province and identified by its biological characters and ITS sequences. All of the strains were cultured on complete medium (CM) [14] at 28 °C for 3–14 days. Equol (Daicel Chiral Technologies Co., LTD., Shanghai, China) was dissolved in methanol (50 mg/mL) and sterilized by filtration as a stock solution. For each experiment, the stock solution was first diluted with methanol into gradient concentrations and then added to treatments to ensure that an equal concentration of methanol background was contained in each treatment.

### 2.2. Assays of Mycelia Growth, Conidial Generation, Germination, and Appressorial Formation

The *M. oryzae* strain Guy-11 in 5-mm-diameter colony plugs was incubated on CM, CM supplemented with equol, daidzein, or 2% methanol on 6-cm culture plates at 28 °C for 6 days. Then, the colonial diameters were measured with a straight ruler. The relative growths were calculated by the ratio of the diameters to that on CM and compared statistically. To calculate the conidia generation, the *M. oryzae* strain Guy-11 was cultured on CM with or without (*S*)-equol on 6-cm culture plates at 28 °C for 6 days. The conidia were washed-off from the colonial surface with sterilized water and a small brush, filtered with three-layer lens cleaning paper, and then counted on a hemocytometer. The conidia generated per unit area were calculated in each treatment and the relative conidia generation to that on CM was compared. For conidial germination and appressorial formation, the conidia harvested from 10-day-old CM plates with similar methods were washed three times and re-suspended at 1 × 10^5^ conidia/mL. Aliquots (50 μL) of the suspensions were incubated on a plastic coverslip in a moist chamber at 28 °C for 24 h. Conidial germination and appressorial formation were examined under a microscope at 2, 4, 6, 8, 12, and 24 h post incubation. To test the restoration of germination, conidia were treated with (*S*)-equol for 2 h, and then eluted with sterilized water three times. The assays for fluorescein diacetate (FDA) staining, Calcofluor staining, and fluorescent microscopy were performed as described previously [15].

### 2.3. Pathogenicity Tests

The conidia harvested from 10-day-old CM plates were re-suspended in 1 × 10^5^ conidia/mL and used for pathogenicity tests. For spray inoculation, rice CO39 was cultured in seedling pots for 14 days, with 20 seedlings per pot. The seedlings were sprayed evenly with the conidial suspension as described previously [14], with 2 mL of suspension for each group of three pots. The inoculated seedlings were incubated in a moist chamber at 28 °C in darkness for 24 h and then in a 12 h light/12 h darkness cycle for 3 days. For the inoculation on detached barley leaves, the barley ZJ-8 was cultured for 7 days and the top first leaves were cut into 5-cm length sections. Then, 20 μL aliquots of the conidial suspension were drop inoculated on the leaf sections. The inoculated leaves were put into 9-cm petri dishes, where three-layers filter paper saturated with sterilized water were used to maintain humidity. The petri dishes were incubated in a moist chamber at 28 °C in darkness for 24 h and then in a 12 h light/12 h darkness cycle for 3 days.

## 3. Results

### 3.1. Equol Inhibits Mycelia Growth and Conidia Generation of M. oryzae

**Figure 1:**
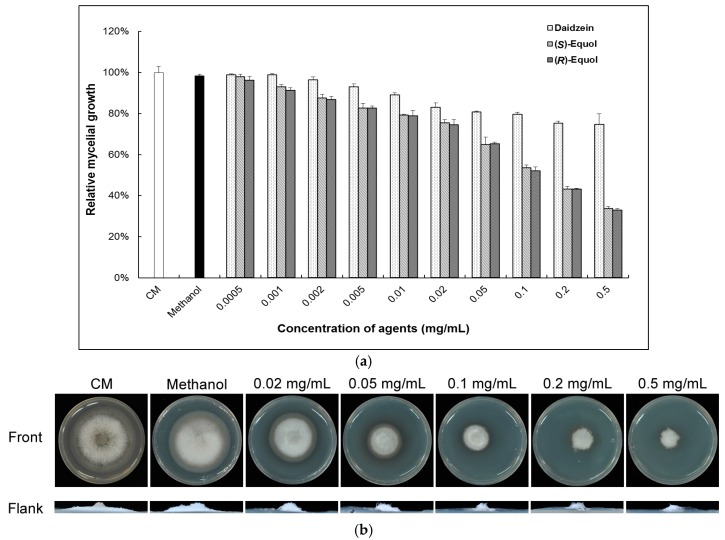

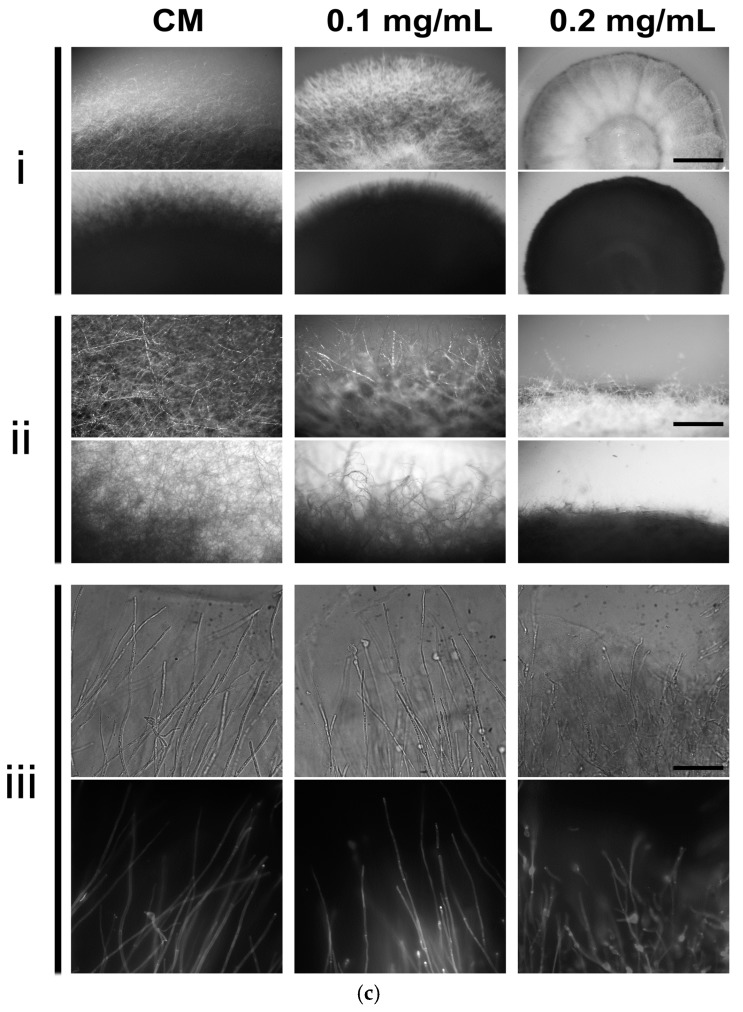
Inhibition test of equol on the mycelial growth of *Magnaporthe oryzae*. The *M. oryzae* strain Guy-11 was cultured on complete medium (CM), CM supplemented with equol or daidzein in gradient concentrations, or with 2% methanol on 6-cm culture plates at 28 °C for 6 days. (**a**) The colonial diameters were measured and the relative growths to that on CM were statistically compared. Error bars represent the deviation calculated from three replicates; (**b**) The images of the colonies on CM and CM with (*S*)-equol; (**c**) The microscopic analysis of the mycelial growth upon (*S*)-equol treatment. i and ii, the colonial edges were observed under a stereoscopic microscope with reflection light (the upper panels) and transmission light (the lower panels). iii, the mycelia at the colonial edges were stained with Calcofluor white and detected under a fluorescence microscope: the upper panel with a bright channel and the lower panel with a fluorescent channel. The bar in i represents 5 mm; in ii, 0.5 mm; and in iii, 5 μm.

The effect of equol on the mycelia growth of *M. oryzae* was tested on CM. In view of that (*S*)-equol can be converted from daidzein in animal gastrointestinal tracts, the daidzein as a comparison was also tested here in the same concentrations. The colonies formed on CM supplemented with equol were found to be significantly smaller than those on CM and CM with a methanol background (Figure 1). The EC_50_ (50% effective concentration) for (*S*)-equol and (*R*)-equol to mycelia growth were calculated as 0.133 ± 0.01 mg/mL and 0.129 ± 0.008 mg/mL, respectively, without significant difference. Although the daidzein also showed inhibition activity to the growth of *M. oryzae*, the inhibition was at much lower levels (EC_50_ = 4.694 ± 0.05 mg/mL) than that of the equol. From light and fluorescent microscopy combined with hyphal staining, we found that the fungus on CM with equol produced much less developed hyphae than those on CM without equol.

**Figure 2:**
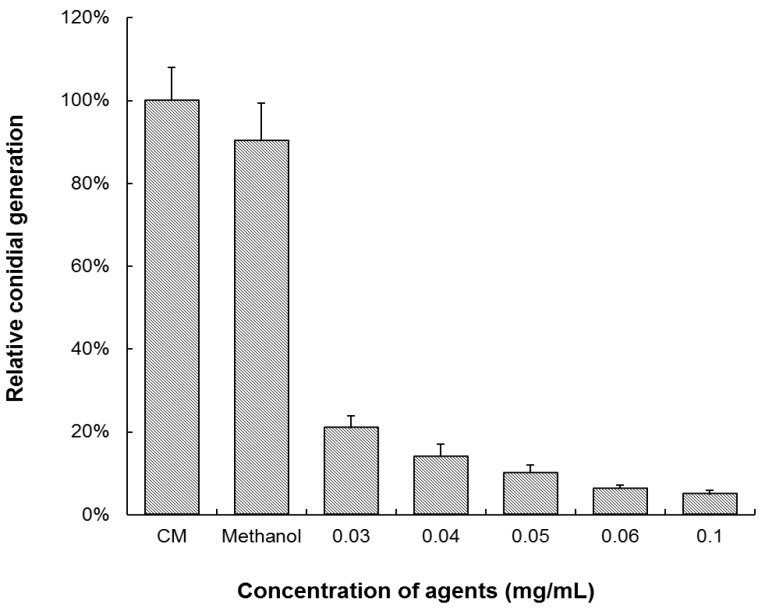
Inhibition test of equol to the conidiation of *Magnaporthe oryzae*. The *M. oryzae* strain Guy-11 was cultured on CM, CM supplemented with (*S*)-equol, and CM with 2% methanol at 28 °C for 6 days. The conidia were harvested from the cultures and counted to calculate the conidiation per square centimeter. The conidiation relative to that on CM was statistically compared. The error bars represent the deviation calculated from three replicates.

Conidia generation was measured on 6-day cultured colonies on CM or CM supplemented with (*S*)-equol. The colonies cultured on CM with (*S*)-equol produced dramatically fewer conidia compared with that on CM and CM with a 2% methanol background (Figure 2). The EC_50_ calculated for the inhibition of (*S*)-equol to the conidiation of *M. oryzae* was 0.0099 ± 0.0002 mg/mL. The results indicated that equol is capable of inhibiting both the growth and the conidiation of *M. oryzae*.

### 3.2. Equol Affects Conidial Germination and Appressorial Development

**Figure 3:**
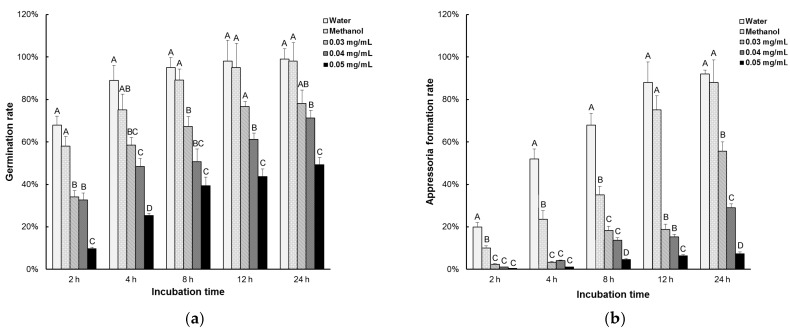
Inhibition test of equol to conidial germination and appressorial formation of *Magnaporthe oryzae*. Conidia harvested from 10-day-old CM plates were re-suspended at 1 × 10^5^ conidia/mL. (*S*)-equol was added in the suspensions to different final concentrations, and methanol was supplemented to 0.2% in each treatment. Aliquots (50 μL) of the suspensions were incubated on plastic coverslips at 28 °C for 24 h. The conidial germination rates (**a**) and appressorial formation rates of the germinated conidia (**b**) were examined at 2, 4, 6, 8, 12, and 24 h post incubation and statistically compared. At least 200 conidia were counted for each treatment. The error bars represent the deviation calculated from three replicates. The capital letters on top of the columns represent the significance of the difference (*p* < 0.01) between the treatments in each time point.

To examine the effect of equol on conidial germination and appressoria development, the conidia suspensions treated with (*S*)-equol or untreated were incubated on a hydrophobic surface and observed under an optical microscope at 2, 4, 8, 12, and 24 h post incubation. Based on the data for the inhibition of equol on mycelial growth and conidiation and on our pre-experiment results, we chose three efficient concentrations (0.03, 0.04, and 0.05 mg/mL) in this experiment. The rates of conidial germination and appressorial formation were both remarkably reduced upon treatment with (*S*)-equol in these three concentrations (Figure 3). The conidia untreated or treated with a 0.2% methanol background were almost fully germinated and formed mature appressoria within 24 h post incubation, while in 0.05 mg/mL (*S*)-equol, only 30% of the conidia germinated and 7% of the generated conidia formed appressoria at 24 h post incubation.

### 3.3. Equol Inhibits Pathogenicity of M. oryzae on Rice and Barley

**Figure 4:**
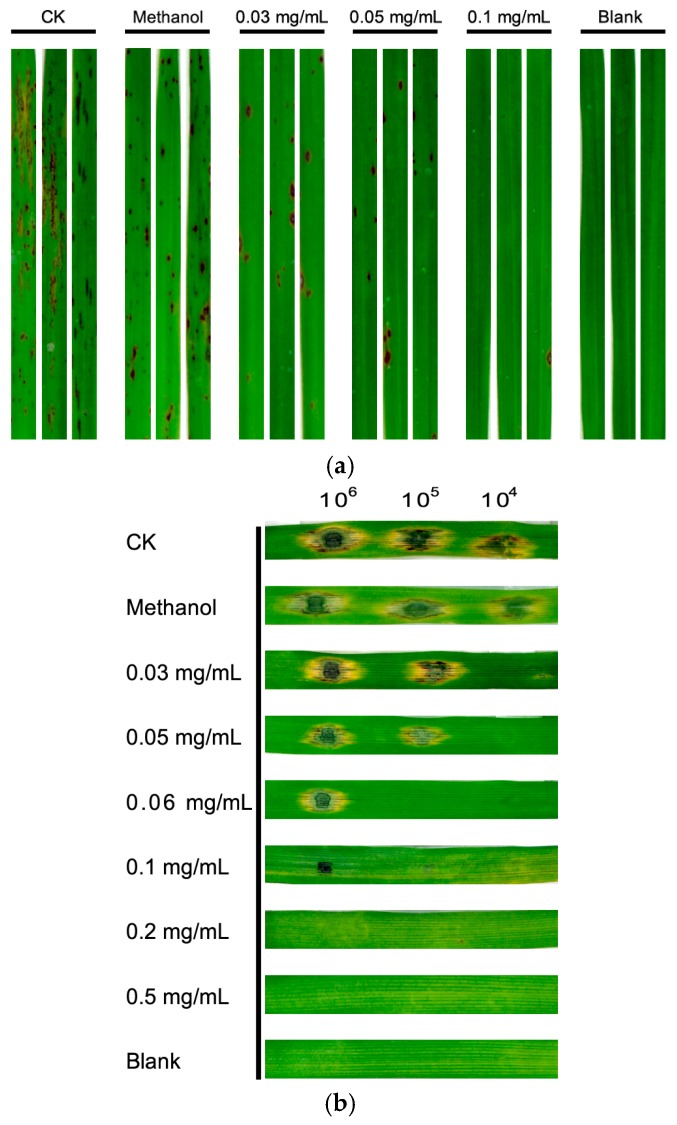
Effects of equol on the pathogenicity of *Magnaporthe oryzae* on rice and barley leaves. Conidia harvested from 10-day-old CM plates were re-suspended at 1 × 10^6^, 1 × 10^5^ and 1 × 10^4^ conidia/mL respectively. (*S*)-equol was added to different final concentrations and methanol was supplemented to 2% in each treatment. (**a**) 14-day-old rice CO39 seedlings were inoculated with the 1 × 10^5^ conidia/mL suspensions (CK) or the suspensions with different concentrations of (*S*)-equol or with a methanol background and incubated at 28 °C in darkness for 24 h and then in a 12 h light/12 h darkness cycle for 6 days; (**b**) Detached leaves of 7-day-old barley ZJ-8 were inoculated with the aliquots (20 μL) of the suspensions with or without (*S*)-equol or with methanol and incubated at 28 °C in darkness for 24 h and then in a 12 h light/12 h darkness cycle for 3 days. The plants used as a blank control were inoculated with sterilized water in the same procedures.

To determine the effects of equol on the pathogenicity of *M. oryzae*, we performed inoculation tests on two hosts, rice and barley. The seedlings of 14-day-old rice cultivar CO39 and detached leaves of 7-day-old barley cultivar ZJ-8 were inoculated by conidial suspensions supplemented with or without (*S*)-equol. (*S*)-equol exhibited a remarkable inhibition of disease development in both hosts (Figure 4). In contrast to the numerous typical lesions on the rice leaves caused by the controls (conidial suspension untreated or that with a methanol background), the symptoms were greatly reduced on the leaves inoculated with conidia treated with (*S*)-equol, or even completely absent upon treatment with 0.1 mg/mL (*S*)-equol. Similar results were obtained in the inoculation test on barley leaves. These results indicated clearly that the equol inhibited the pathogenicity of the fungus.

### 3.4. Effects of Equol Are Restorable

**Figure 5:**
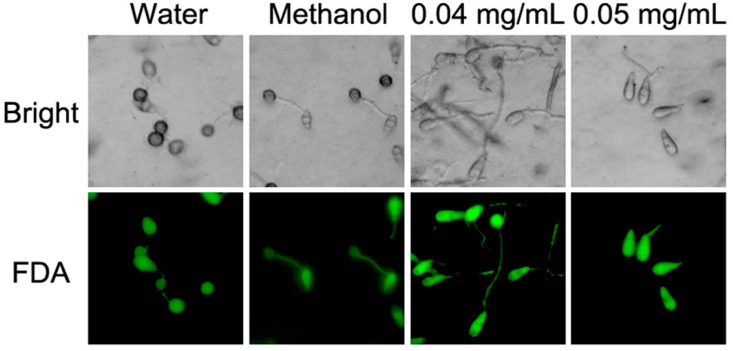
FDA staining to test the viability of conidia treated with equol. Conidia suspensions (1 × 10^5^ conidia/mL) supplemented with 0.04 mg/mL and 0.05 mg/mL (*S*)-equol were incubated on plastic coverslips at 28 °C in darkness for 24 h to allow for germination and appressorial formation. The suspensions without (*S*)-equol and that containing 0.2% methanol were used as controls. After incubation for 24 h, the conidia and appressoria were stained with 100 μg/mL FDA solution for 5 min and detected under a fluorescent microscope.

To know whether the conidia were killed by equol treatment, we tested the cell viability using an FDA staining assay. However, the conidia treated with (*S*)-equol were found to be still alive. Even when treated for 24 h, more than 98% of the conidia adapted to the FDA staining and emitted green fluorescence (Figure 5).

**Figure 6:**
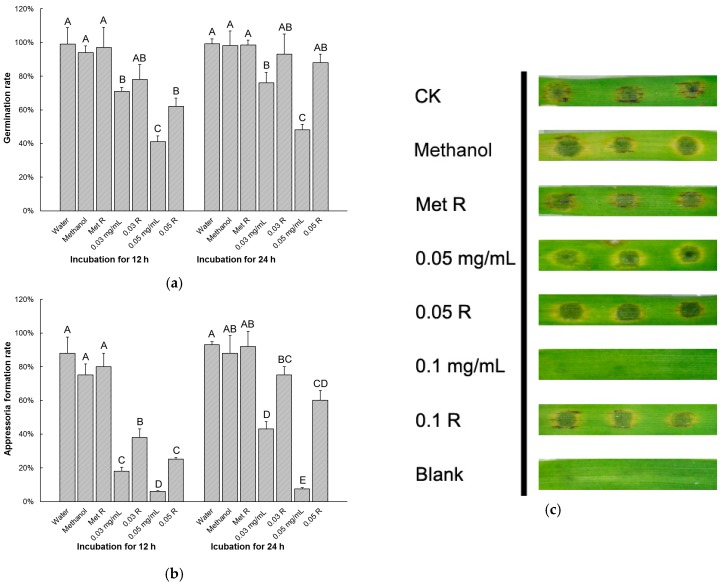
The inhibition of equol to conidial germination, appressorial formation, and pathogenicity is restorable by removing the equol. The conidia suspensions (1 × 10^5^ conidia/mL) that were supplemented with 0.03 and 0.05 mg/mL (*S*)-equol, and those that were treated with 0.03 and 0.05 mg/mL (*S*)-equol for 2 h then washed in sterilized water three times (0.03 R and 0.05 R) were incubated on a plastic coverslip at 28 °C for 24 h to allow for germination and appressorial formation. Conidial germination (**a**) and appressorial formation (**b**) at 12 and 24 h post incubation were examined and statistically compared. The error bars represent the deviation calculated from three replicates. The letters in capital on top of the columns represent the significance of the difference (*p* < 0.01) between the treatments in each time point; (**c**) The conidia suspensions (1 × 10^5^ conidia/mL) that were supplemented with 0.05 and 0.1 mg/mL (*S*)-equol, and those that were treated with 0.05 and 0.1 mg/mL (*S*)-equol for 2 h and then washed in sterilized water three times (0.05 R and 0.1 R) were inoculated on 7-day-old barley ZJ-8 leaves and then incubated at 28 °C in darkness for 24 h and a subsequent 12 h light/12 h darkness cycle for 3 days. Conidia suspensions that were untreated (CK), treated with 0.2% methanol, and treated with 0.2% methanol for 2 h then washed with sterilized water (Met R) in the same procedures were used as controls. The plants inoculated with sterilized water were used as a blank control.

We then investigated whether the effects of equol on conidia are revertible by removing the equol from the suspension. The ability of the conidia to germinate and form appressoria was found to be partially restored when the conidia treated with (*S*)-equol for 2 h were washed with water (Figure 6a). The pathogenicity of the conidia on barley was also regained to a large extent when the conidia were released from a 2-h (*S*)-equol treatment by rinsing with water (Figure 6). These results, according with that the conidia are still alive after equol treatment, indicate that the equol inhibits conidia from germinating and forming appressoria rather than killing them.

### 3.5. Antifungal Activity of Equol Is Not Related to Peroxisome

**Figure 7:**
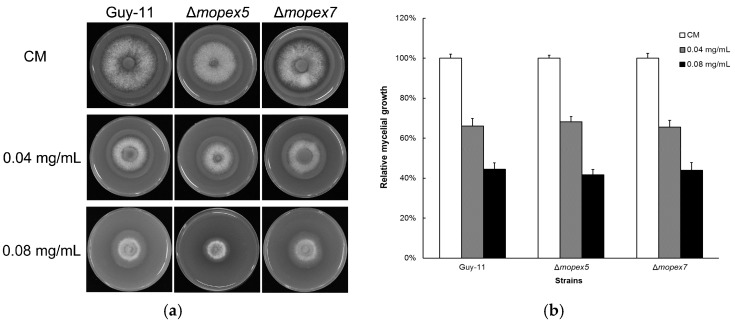
Comparison of the inhibition of equol to the mycelial growth of *Magnaporthe oryzae* wild-type Guy-11 and the Δ*mopex5* and Δ*mopex7* mutants. (**a**) The strains were cultured on CM and CM supplemented with 0.04 mg/mL and 0.08 mg/mL (*S*)-equol at 28 °C for 6 days on 6-cm culture plates; (**b**) the colonial diameters of the strains were measured and the relative growth was calculated and compared. The error bars represent the deviation calculated from three replicates.

In mammals, daidzein can influence target cells by transactivating three peroxisomal proliferation activator receptors (PPARs) α, δ, and γ [17]. To test whether the effects of equol to *M. oryzae* are related to peroxisome, we compared the mycelial growth of wild-type Guy-11 and two mutants defective in peroxisomal biogenesis on (*S*)-equol-containing CM. Our data showed that the *M. oryzae PEX5* gene-deleted mutant (Δ*mopex5*) and the *PEX7* deleted mutant (Δ*mopex7*) [15] were inhibited by (*S*)-equol in equivalent levels to the wild-type (Figure 7). These findings may suggest that the antifungal activity of equol to *M. oryzae* is not related to the peroxisome.

### 3.6. Antifungal Activity of Equol on Other Fungal Pathogens

**Figure 8:**
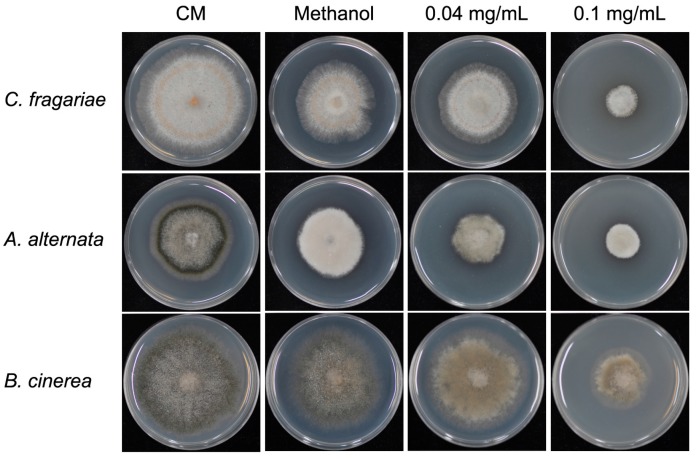
Inhibition test of equol to the mycelial growth of *Colletotrichum fragariae*, *Alternaria alternata*, and *Botrytis cinerea*. The strains were cultured on CM, CM supplemented with 0.04 and 0.1 mg/mL (*S*)-equol, or with 2% methanol at 28 °C for 6 days on 6-cm culture plates.

To further reveal whether the antifungal activity of equol is broad-spectrum to fungal pathogens, we assessed the inhibition of (*S*)-equol to the vegetative growth of another three important pathogenic fungal species, *Colletotrichum fragariae*, *Alternaria alternata*, and *Botrytis cinerea*. Analogously to that observed in *M. oryzae*, the (*S*)-equol effectively inhibited the mycelial growth of all of the three fungi (Figure 8).

## 4. Discussion

Rice is the most important crop in Asia and supplies food for more than half of the global human population. Rice blast is the most destructive disease influencing rice production and food safety in almost all of the rice-growing regions. For the control of rice blast, massive amounts of chemical fungicides are applied every year. The widespread use of synthetic fungicides causes problems such as the emergence of resistant pathogens, residual toxicity, and environmental pollution [18,19]. To solve these problems, scientists have been actively seeking natural products and natural product-derived metabolites as potential fungicidal substances, since natural products are generally considered to have low mammalian toxicity and to be degradable in environments [20]. In this study, we firstly demonstrated that equol, a metabolite from natural soy isoflavone, has antifungal activities against rice blast pathogen *M. oryzae*, and the activities involve inhibition to not only mycelial growth, but also to conidia generation, conidial germination, appressorial formation, and pathogenicity.

As naturally occurring substances, isoflavonoids have been closely focused on for their biological properties [21], mainly because of their potential association with anticancer and human health [9,22] as well their potential fungicidal activity. The effects of isoflavonoids on fungal growth have been investigated in several fungal species [23,24,25,26,27,28] and the antifungal factors in certain plants, such as red clover and infected soya, are regarded as isoflavonoids [29,30,31]. Naim et al. have reported that free isoflavones possess depression activity to the growth of *Trichoderma lignorum*, *Rhizoctonia solani*, *Fusarium oxysporum*, *Pythium* spp, *Rhizopus* spp, and *Sclerotium rolfsii*, while the antifungal activity of isoflavones glycosides was negligible in most instances [32]. Weidenbörner et al. compared the effects of two naturally occurring isoflavones, genistein and biochanin A, and their derived isoflavanones and isoflavans on the mycelial growth of two soil-borne fungi *R. solani* and *S. rolfsii* [33], and indicated that all of the isoflavonoids of the biochanin A series showed high antifungal activity, genistein isoflavan and the other isoflavans with two hydroxyl groups and one methoxy group were fungi toxic, while isoflavans with two or three methoxy groups were almost inactive. Krämer et al. tested the antifungal activity of the isoflavones from soybean (*Glycine max*) and chickpea (*Cicer arietinum*) and their derived isoflavanones and isoflavans on three food- and forage-containing fungi, *Aspergillus ochraceus*, *Penicillium digitatum*, and *Fusarium culmorum* and found that these compounds were variable in their activity, either as a stimulator or as an inhibitor, to fungal growth due to the variation of concentration and fungal targets [23]. Accordingly, the isoflavans, although acting as inhibitors in most cases, stimulated the growth of *P*. *digitatum* and *F*. *culmorum* at certain concentrations. Metabolized from soy isoflavone, equol is thus an isoflavan in structure [2,3]. Our data showed that the equol functioned consistently as an inhibitor at all of the tested concentrations. These studies indicated that the fungicidal property of the isoflavonoids is related largely to their composition and structures, and vary greatly on different target fungi.

Kasugamycin and tricyclazole are two of the most commonly used chemical agents for the control of rice blast [34,35]. The biochemical action of kasugamycin is to inhibit protein synthesis [36]. Protein synthesis inhibitors generally have a higher activity on mycelial growth than conidial germination [37]. Correspondingly, kasugamycin was active to mycelial growth, with a 56% inhibition rate at 50 μg/mL, but was not efficient enough to inhibit the conidial germination of *M*. *oryzae* [38]. The EC_50_ of equol to mycelia growth is more than 100 μg/mL, while 30 to 50 μg/mL equol inhibited conidial germination and appressoria formation efficiently, indicating that equol, in contrast to Kasugamycin, has a relatively strong inhibitory effect to conidial germination and appressoria formation than to mycelia growth. Tricyclazole specifically inhibits melanin synthesis in the rice blast fungus and thus affects the function of appressoria which deposit melanin on cell walls to maintain a high internal turgor pressure [39,40]. Therefore, tricyclazole at low concentrations (1 μg/mL) did not inhibit vegetative growth but was capable of controlling the rice blast disease [41]. However, in our data, equol can inhibit growth, conidiation, conidial germination, and appressorial formation. In addition, based on mycelium growth, the EC_50_ of a collection of rice blast isolates in Australia were 0.02 to 2.02 µg/mL for azoxystrobin and 0.06 to 1.91 µg/mL for propiconazole [42]. The EC_50_ for azoxystrobin and kresoxim-methyl were 0.006 to 0.056 and 0.024 to 0.287 µg/mL, respectively, in inhibiting mycelial growth of 80 *M. oryzae* isolates in Anhui Province of China [43]. In spite of the fact that the fungal strains and the test assays are different in these studies, the inhibition efficiency of equol to mycelia growth is quite weak compared with these chemicals. However, interestingly, the inhibitions of equol to conidiation and to appressorial formation appear to be relatively strong, which come up to or exceed the levels of the above chemicals. This data may suggest that equol acts via a different mode in its antifungal activity to those of kasugamycin, tricyclazole, and the commonly used chemicals.

In mammalians, daidzein and (*S*)-equol were found to act as agonists of the G protein-coupled estrogen receptor GPER/GPR30 [44]. Equol exists in two enantiomeric forms (*S*)-equol and (*R*)-equol, among which only (*S*)-equol is produced in humans and animals [2]. The molecular and physical structure of (*S*)-equol is similar to that of the hormone estradiol [45], and (*S*)-equol preferentially binds estrogen receptor beta [3,46]. The bioactivity of daidzein has thus been thought to associate with its ability to produce (*S*)-equol. Accordingly, (*S*)-equol exhibited better bioactivity than (*R*)-equol and daidzein [46]. In the present study, equol has higher antifungal activity than daidzein; however, (*R*)-equol and (*S*)-equol did not show differences in their ability to inhibit fungal growth. Additionally, in the *M*. *oryzae* genome, we failed to find homologous proteins to the mammalian GPER/GPR30. Another action of daidzein is to transactivate three peroxisomal proliferation activator receptor (PPAR) isoforms, α, δ, and γ, and influence target cells [47]. Our data showed that the *M*. *oryzae* mutants defective in peroxisomal biogenesis [15] have an equivalent sensitivity to equol as the wild-type. These findings indicate that the action mechanism in the antifungal activity of equol to *M*. *oryzae* is likely distinct from that in the other bioactivity to mammalian cells. There is still insufficient evidence as to whether equol activates the G protein-coupled receptor in *M*. *oryzae*, and to reveal the action mechanism of equol in fungicidal activity, more investigations will need to be done in the future.

The ability to transform daidzein into (*S*)-equol in humans is based on the presence of certain intestinal bacteria. Studies indicate that 25–30% of the adults in Western countries, and 50–60% of the adults from Japan, Korea, or China produce (*S*)-equol after eating isoflavone-containing foods [4,45,48,49,50,51,52,53]. In very recent years, the research on intestinal microflora has attracted abundant attention and has become a hot topic worldwide [54,55]. This research prompted the isolation of the equol-producing bacteria or mixed microbial cultures [56,57]. Isolating such bacteria and testing their antifungal activities on fungal pathogens may allow us to find new potential biocontrol agents for plant diseases. Identifying the genes involved in equol production in these bacteria may provide new gene choices for the breeding of transgenic disease-resistant plants.

## 5. Conclusions

In summary, equol, the metabolite of daidzein from mammalian intestinal bacteria, can negatively affect the mycelial growth, conidiation, conidial germination, appressoria formation, and pathogenicity of the rice blast fungus, *M. oryzae*, and also has the potential to inhibit the development of other plant fungal pathogens. These findings expand our knowledge on the bioactivities of equol and enlighten us on plant disease control.
